# Evaluating the Effectiveness of Instructional Videos as a Tool for Teaching Orthopaedic Casting Technique: A Randomized Study Among Undergraduate Medical Students

**DOI:** 10.7759/cureus.79825

**Published:** 2025-02-28

**Authors:** Azlina Amir Abbas, Kwong Weng Loh, Mohd Reza Mohd Aridz, Khairul Anwar Ayob

**Affiliations:** 1 Department of Orthopaedic Surgery, Universiti Malaya, Kuala Lumpur, MYS; 2 Department of Orthopaedic Surgery, Universiti Teknologi MARA, Sungai Buloh, MYS

**Keywords:** education and training of medical students and doctors (specialist and phd), instructional methods in health professions education, medical student training, orthotic, video-based learning

## Abstract

Background

Casting and splinting are crucial components of fracture management, making it essential for medical students to acquire proper application skills during their orthopaedic rotations. In this study, the authors aimed to investigate the potential enhancement of skill acquisition through the inclusion of an instructional video.

Methods

The study was conducted at a tertiary education centre in Malaysia in July 2015, and a convenience sampling of final-year medical students was used. The sample consisted of 108 students who were assigned into three groups. While all participants received a lecture on cast application, one group was provided with a sequential instructional video, and another group was guided using a segmented video. Subsequently, all participants were tasked with applying an above-elbow cast, and their performance was evaluated using a 14-item Objective Structured Clinical Examination (OSCE) score. Descriptive statistics were computed for all baseline demographic variables. To compare the scores among the different groups, a one-way analysis of variance was conducted. The correlation between the OSCE score and the teaching method was assessed using the Pearson correlation coefficient.

Results

The mean age of medical students was 23 years, and 70 (64.8%) of the students were female. The mean OSCE scores were significantly higher in students exposed to instructional videos (group 2: 8.21 ± 0.92; group 3: 8.35 ± 0.86) compared to those who received only a lecture (group 1: 7.49 ± 1.03, p < 0.001). After adjusting for potential confounding factors, the teaching method emerged as the only significant predictor influencing OSCE scores. There was a positive correlation between the teaching method and the total OSCE score, with a correlation coefficient of 0.352 and p < 0.001.

Conclusions

The addition of instructional video teaching had a significant positive impact on the performance of above-elbow full cast application compared to relying solely on didactic teaching. Both sequential and segmented video demonstrations were effective in improving the outcomes of the technique.

## Introduction

Orthopaedic casting and splinting are fundamental clinical skills in orthopaedic surgery, serving as the preferred method for immobilization and pain management in acute cases, as well as the primary treatment approach for most fractures [1]. However, despite the significant emphasis on learning plaster of Paris (POP) cast application during medical school, medical students and house officers often express a lack of confidence and proficiency in this technical skill, leading to growing concerns about the quality of patient care, and increased risk of complications [2-5].

Mastering POP cast and splint application requires practice and a strong grasp of casting principles. A basic understanding of musculoskeletal medicine is also essential. However, the limited duration of clinical attachments in orthopaedic surgery may not provide sufficient time for students to acquire this vital skill. Additionally, the emphasis on musculoskeletal medicine within medical schools is often inadequate, failing to ensure that medical students attain basic competence in this subject [2,5-7]. Consequently, there is a need to revamp the teaching strategies employed to address this challenge.

There is a lack of studies assessing the effectiveness of instructional videos in helping students acquire the skills of orthopaedic casting. We wish to utilize educational videos to showcase casting techniques with comprehensive, step-by-step guidance. We believe this approach enables students to review the videos at their convenience, aiding in the learning and mastery of various skills. Research has indicated that the use of instructional videos has resulted in improved clinical skills among students when compared to traditional teaching methods [8-11]. In a study by Mehrpour et al., it was suggested that a supplemental video instructional program enhanced students' performance in musculoskeletal clinical skills, surpassing the outcomes achieved through traditional lectures alone. The study further emphasized that these videos could serve as valuable tools for enhancing splinting skills [12]. While prior studies have demonstrated the effectiveness of video-based teaching in clinical education, limited research has specifically compared segmented versus sequential instructional video formats in orthopaedic casting training.

Given these challenges, this study seeks to systematically evaluate the impact of different instructional video formats on student learning and confidence in orthopaedic casting. This study aims to compare the effectiveness of segmented versus sequential instructional videos in improving orthopaedic casting skills among undergraduate medical students, addressing the gap in structured video-based training for procedural skills.

## Materials and methods

The study was conducted at a tertiary education centre in Malaysia, and a convenience sampling of final-year medical students was used. Each student received a detailed participant information sheet, and the research adhered to the principles outlined in the Helsinki Declaration. Ethical approval was granted by the university. Informed consent was obtained from all participants.

**Figure 1:**
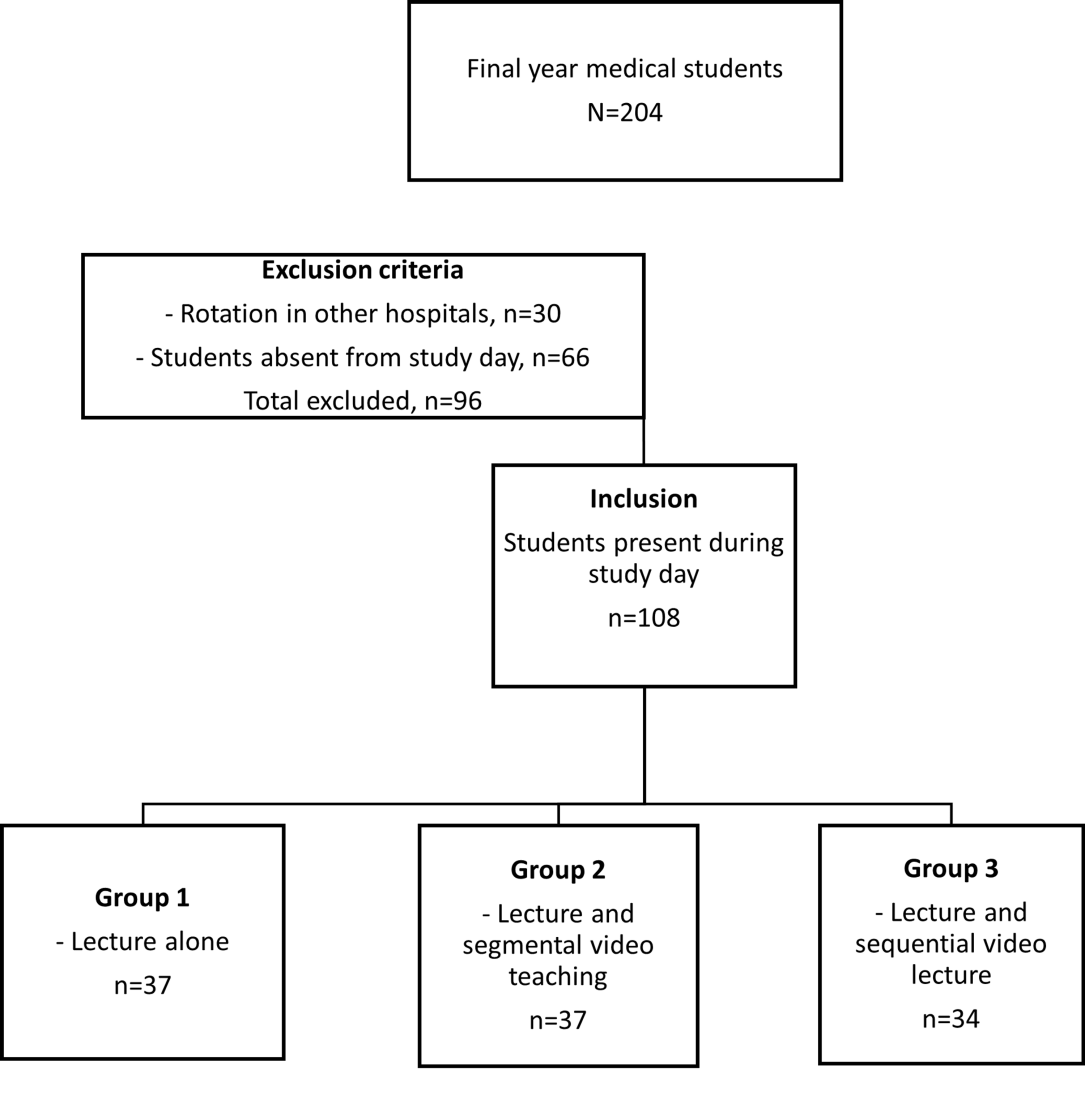
CONSORT diagram of the study. CONSORT: Consolidated Standards of Reporting Trials.

A total of 108 students were enrolled and randomly divided into three groups. Randomization was conducted using a tricolour-coded participant information sheet distributed sequentially as students entered the lecture theatre, ensuring an unbiased allocation process. Group 1, with 37 students, received only the lecture; group 2, with 37 students, received the lecture followed by a segmented video presentation; and group 3, with 34 students, received the lecture followed by a sequential video presentation (Figure 1). Participants were expected to perform an above-elbow cast application at the end of their teaching session.

All participants received a PowerPoint (Microsoft Corporation, Redmond, WA) lecture presentation on the technique of applying an above-elbow full cast. The lecture was delivered by a senior registrar in orthopaedic surgery and comprised 28 slides. The presentation covered the objectives of a POP cast application, the purpose of immobilization, complications associated with casting, and the technique for applying an above-elbow full cast. Following the lecture, participants were divided into their respective groups. Group 1 proceeded directly to the above-elbow full cast application task. Group 2 was required to perform the task while watching the instructional video, which was played in segments, as this approach allows for better retention of information, facilitates step-by-step learning, and enables learners to focus on specific techniques without feeling overwhelmed. Group 3 watched the instructional video in its entirety and then completed the task.

The study utilized a five-minute instructional video that was created by two of the authors. The video featured a comprehensive demonstration of the step-by-step process of plaster application on the upper limb of a simulated patient conducted by an expert. The five-minute video was divided into three segments: (1) preparation and materials (one minute); (2) step-by-step cast application (three minutes and 15 seconds); and (3) post-application assessment (45 seconds). Each individual step was explained in detail, and essential information, such as the materials and equipment used, was highlighted. Following the video, each student had the opportunity to apply a plaster cast on a simulated patient. A total of 14 skills in the casting of an injured limb were evaluated (Table 1). The assessment was carried out by four senior registrars in orthopaedics.

**Table 1:** Question and scoring for casting.

You are attending to a patient with an undisplaced distal end radius fracture, and you decide to treat him/her conservatively with above-elbow full-length plaster of Paris (POP)		
	Checklist	Score
1.	Universal precaution: put on gloves and apron	0.5
2.	Patient notification: introduce yourself and explain the procedure	0.5 0.5
3.	Positioning of the limb	0.5
4.	Joints should be placed in proper position, wrist in resting neutral position, forearm semi pronated, elbow 90° flexion	1
5.	Application of stockinette	0.5
6.	Application of softban with the appropriate amount of padding, especially at the bony prominences	0.5
7.	Submerge dry roll of POP in water until bubbling material stops and gently squeeze excess water	0.5
8.	Roll out POP from distal to proximal with appropriate layers, in correct wrist position and moulded to contours of the hand and wrist	1
9.	Roll out POP from distal to proximal with appropriate layers, in correct forearm and elbow position across the elbow and moulded to contours of the limb	1
10.	Roll out POP from distal to proximal with appropriate layers, in correct wrist, forearm and elbow position and moulded to contours of the limb	1
11.	Thumb free and distal cast till distal palmar crease and proximal to the metacarpophalangeal (MCP) joints and the patient able to perform grip	1
12.	Absence of finger crowding	0.5
13.	No cast tightness or numbness, distal circulation <2 seconds	0.5
14.	Smooth the edge and surface of the cast	0.5

The competency, knowledge, and skills of the medical students were assessed using an Objective Structured Clinical Examination (OSCE) score sheet [13]. This 14-item score sheet was developed based on the steps involved in applying the cast and was created using a previously validated questionnaire [12]. Each step in the cast application process was assigned a score ranging from 0.5 to 1, with a higher overall score indicating better performance. Additionally, a global rating scale was used to assess the appearance and functionality of the cast applied. The internal consistency of the 14-item OSCE overall performance scale was deemed acceptable, yielding a Cronbach's alpha of 0.7. The Cronbach's alpha value reflects the relationship between the number of items in the OSCE scale and the mean summed scores of those items, indicating the level of technical skill performance [14].

The OSCE checklist was pilot-tested for face and content validity by four senior orthopaedic lecturers before implementation. Following the completion of the POP application procedure, students were required to fill in a satisfaction of learning score sheet (Table 2). Simulated patients also provided feedback by completing a patient satisfaction score sheet (Table 3). Both score sheets were based on a five-point Likert scale adapted from a review of relevant literature [15].

**Table 2:** Satisfaction of learning. POP: plaster of Paris.

Satisfaction of learning scale	Five-point Likert scale				
	Strongly agree	Agree	Neither agree nor disagree	Disagree	Strongly disagree
The teaching process was excellent	5	4	3	2	1
I know more about POP and POP application and dangers	5	4	3	2	1
I can confidently apply my next POP	5	4	3	2	1
I am satisfied with my learning progress today	5	4	3	2	1
I will be able to remember and recall my learning today in three months	5	4	3	2	1

**Table 3:** Patient satisfaction.

Patient satisfaction scale	Five-point Likert scale				
	Strongly agree	Agree	Neither agree nor disagree	Disagree	Strongly disagree
I was well-informed regarding the procedure	5	4	3	2	1
The doctor appears confident and well prepared	5	4	3	2	1
The application process was smooth and done step by step	5	4	3	2	1
The doctor was very gentle and tidy	5	4	3	2	1
I am happy and satisfied with my cast	5	4	3	2	1

Statistical analyses

All data were stored, structured, and analysed using SPSS software for Windows, version 23 (IBM Corp., Armonk, NY). Descriptive statistics were computed for all baseline demographic variables, providing a summary of the characteristics of the participants. To compare the scores among the different groups, a one-way analysis of variance (ANOVA) was conducted. Before conducting ANOVA, data normality was assessed using the Shapiro-Wilk test, confirming assumptions for parametric testing. The correlation between the OSCE score and the teaching method was assessed using the Pearson correlation coefficient. Additionally, a linear multivariable regression model was employed to examine various factors that might influence the students' OSCE scores, with the unstandardized coefficient (β) utilized to determine the effect size of each covariate [16]. All reported p-values were two-tailed, and a significance level of 0.05 was used as the cutoff point for statistical significance.

## Results

A total of 108 medical students were randomly assigned into three separate groups. Groups 1 and 2 comprised 37 students, whereas group 3 had 34 students. The mean age of medical students at the time of the study was 23 years (range: 23-24), wherein 70 (64.8%) of the students were female. A total of 108 simulated patients (consisting of medical students and staff nurses) were also assigned into three groups to match the numbers in the student groups. The overall median age of patients was 20 years (range: 18-23), with 80 (74.1%) being female.

The intraclass correlation coefficient (ICC) for inter-rater reliability during the pretest was 0.81 (CI: 0.78-0.84; p = 0.005), whereas the internal consistency of the 14-item OSCE performance scale during the pretest had an excellent Cronbach’s alpha (α) of 0.93. The students’ satisfaction of learning scale and patient satisfaction scale had an “excellent” (α = 0.90) and “good” (α = 0.85) reliability and internal consistency, respectively.

**Figure 2:**
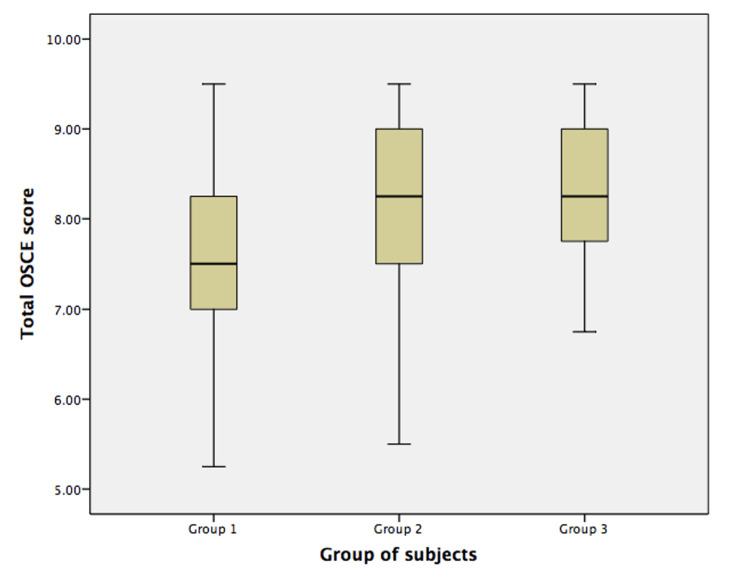
The Objective Structured Clinical Examination (OSCE) score comparisons among groups.

Group 3 students had the highest mean OSCE score (8.35 ± 0.86), followed by group 2 students (8.21 ± 0.92) and group 1 students (7.49 ± 1.03) (Figure 2). Analysis of variance showed significant between-group differences (p < 0.001). Post-hoc Tukey analyses revealed groups with instructional video presentation (groups 2 and 3) had statistically significantly higher mean scores when compared to group 1 (p < 0.005 and p < 0.001, respectively) (Table 4). After adjusting for potential confounders, the teaching method is identified as the sole significant predictor that affected OSCE scores (Table 5). There is a positive relationship between teaching method and total OSCE score (r = 0.352, p < 0.001). The observed improvement of approximately 10% in OSCE scores among students exposed to instructional videos suggests a meaningful enhancement in procedural competence, potentially translating to better patient care.

**Table 4:** Comparison of the Objective Structured Clinical Examination (OSCE) performance scores between groups (n = 108). One-way ANOVA was applied, followed by a post-hoc multiple comparison test using Tukey's procedures. * Statistical significance was defined at p < 0.05.

Comparison group	Mean difference (95% confidence interval)	p-value
Group 1 & group 2	-0.729 (-1.253, -0.206)	0.004*
Group 1 & group 3	-0.866 (-1.401, -0.331)	0.001*
Group 2 & group 3	-0.136 (-0.671, 0.398)	0.816

**Table 5:** Unstandardized coefficient (β), 95% confidence interval, and p-value from linear multivariable regression model with students’ OSCE scores as the dependent variable. POP: plaster of Paris; OSCE: Objective Structured Clinical Examination. * Statistical significance was defined at p < 0.05.

Independent variable	Unstandardized coefficient (β)	95% confidence interval	p-value
Gender	-0.117	-0.506 to 0.271	0.551
Age (years)	0.307	-0.433 to 1.047	0.412
Previous orthopaedics experience	0.064	-0.451 to 0.579	0.806
Previous POP teaching	-0.49	-0.447 to 0.349	0.808
Previous POP application	0.283	-0.389 to 0.955	0.406
Teaching method	0.438	-0.213 to 0.662	<0.001*

## Discussion

Casting and splinting are fundamental skills in the field of orthopaedics and traumatology, serving as the cornerstone of outpatient fracture management. Properly applied casts and splints help stabilize fractures, alleviate discomfort, and contribute to optimal recovery. Medical students must understand the underlying principles of fracture management, anatomy, biomechanics, and materials used in casting. They need to develop the ability to assess and diagnose fractures accurately, select the appropriate type of cast or splint, and apply it skilfully to ensure proper immobilization and patient comfort. However, medical education often faces challenges in effectively teaching these skills due to time constraints, limited exposure to practical training, and varying levels of hands-on experience. To address these challenges, our study aims to explore innovative ways to facilitate medical students in acquiring and mastering the skillset of casting and splinting. Enhanced competence in orthopaedic casting techniques may contribute to reduced complications such as pressure sores, improper immobilization, and unnecessary cast removals, ultimately improving patient outcomes.

In this study, we found that implementing instructional video teaching enhances clinical skills outcome in medical students in POP cast application. We reported higher mean OSCE scores (8.21 and 8.35 vs. 7.49, p < 0.001) when students were exposed to either segmental or sequential video teaching compared to lecture alone. These findings concur with previous literature that showed improvements following video-based education intervention [8,10,17-19]. We believed that video-based education allowed the demonstration of complex procedures and the actual skills needed in the sequence of events, which otherwise may not be adequately represented through lectures only. Our study showed no significant comparison among those who watched the instructional video. The video, presented in a segmented or a sequential manner, had an equally positive impact on the learning outcome. The result was similar to that of Schittek Janda et al., who reported an equally good learning outcome in surgical hand washing following a segmented or sequential instructional video [20].

We found no significant correlation between traditional lectures and the two modes of instructional videos concerning patients' satisfaction. The total average scores among the three groups did not exhibit statistically significant differences. Patients expressed equal levels of satisfaction with both the casts applied and the manner in which they were applied, regardless of how their participants were taught. Patients rated their participants highly in terms of being gentle and maintaining a tidy approach during the application of POP casts, with high average scores observed across all three groups. However, there was a slight difference in patients' perception of participant’s confidence. Patients perceived participants who had been exposed to the instructional videos as being slightly more confident than those who had not. This suggests that the use of instructional videos may have had a positive impact on participants' perceived confidence levels.

Some studies advocate the involvement of a professional instructor. They reported that instructional videos, with the involvement of an instructional assistant, enhance students’ experience and demonstrate better outcomes in clinical skills teaching [21,22]. Not everyone agrees with the result. Xeroulis et al. demonstrated that computer-based video instruction (CBVI) is as effective as summary expert feedback in teaching basic technical skills, such as suturing and instrument knot-tying, to medical students. Similarly, Nousiainen et al. conducted a study involving medical students and found comparable results. They continued to show support for video instruction and instructional assistants to be supplemented together [23,24]. To address this in future studies, we should demonstrate the involvement of instructional assistants as a comparison against the other groups involved.

We acknowledge some limitations in our study, including the relatively small sample size of final-year medical students from our institution. The inclusion of students at different stages of rotations, with a subset (22 students, 20%) having completed or currently undergoing an orthopaedic rotation, may introduce bias. However, our findings indicate that previous orthopaedic experience and knowledge of POP did not significantly impact the OSCE scores (Table 5). The lack of impact from prior orthopaedic experience on OSCE scores suggests that instructional videos may serve as an equalizing factor in skill acquisition, potentially benefiting students regardless of their initial knowledge level. These limitations highlight the need for caution in generalizing the results to a broader population. Every participant was evaluated by a single assessor, with four assessors involved. Therefore, the performance scoring was based on one person’s evaluation, not a mean evaluation of all four assessors, and because of that, a Bland-Altman analysis to assess the inter-assessor agreement was not able to be carried out. Despite this, the ICC for inter-rater reliability during pretest and test-retest was 0.811 (CI: 0.295-0.995; p = 0.005) and 0.833 (CI: 0.401-0.995; p < 0.005), respectively, with the internal consistency of the OSCE score showing excellent Cronbach’s alpha (α) of 0.93 and 0.952, respectively. Inter-rater reliability during test-retest was 0.709 (CI: 0.256-0.906; p < 0.005), with a Cronbach’s alpha (α) of 0.829.

Future research with larger and more diverse samples, while controlling for potential confounders such as previous orthopaedic or casting experience, would help validate and expand upon our findings. Subsequent studies could include follow-up OSCEs or clinical performance assessments weeks or months later to assess skill retention. The insights gained from our study provide valuable information on teaching methods for casting and splinting skills among medical students, highlighting the significance of instructional video to enhance training and build confidence in this crucial aspect of orthopaedic practice. Our research aims to identify the most effective strategies that can be integrated into medical curricula, ensuring that future orthopaedic surgeons and healthcare professionals possess the necessary skills to provide optimal fracture care. By improving education and training in casting and splinting, we can enhance patient outcomes, reduce complications, and contribute to the overall quality of orthopaedic and traumatology practice. The incorporation of instructional videos can offer opportunities for repeated practice, reinforcement of key concepts, and feedback, thereby fostering greater confidence and competence in cast application.

## Conclusions

Our findings indicate that incorporating instructional videos into orthopaedic curricula effectively enhances casting skills. Future research should investigate the long-term retention of these skills and assess the impact of integrating instructor feedback with video-based learning. Additionally, studies are needed to determine whether repeated exposure to instructional videos across multiple training sessions leads to improved retention and sustained competence in casting techniques.
